# Silver ions cause oscillation of bacterial length of *Escherichia coli*

**DOI:** 10.1038/s41598-019-48113-4

**Published:** 2019-08-13

**Authors:** Venkata Rao Krishnamurthi, Jingyi Chen, Yong Wang

**Affiliations:** 1 Department of Physics, University of Arkansas, Fayetteville, AR 72701 USA; 2 Department of Chemistry and Biochemistry, University of Arkansas, Fayetteville, AR 72701 USA; 3 Microelectronics-Photonics Graduate Program, University of Arkansas, Fayetteville, AR 72701 USA; 4 Cell and Molecular Biology Program, University of Arkansas, Fayetteville, AR 72701 USA

**Keywords:** Antimicrobial resistance, Biological physics

## Abstract

Silver (Ag) in various forms have recently gained broad interest and been revisited due to their promising antimicrobial effects. Here we report our study on the morphological dynamics of live bacteria when subjected to Ag^+^ ions. Using time-lapse microscopy, we observed oscillations of cell-length for a large fraction of bacteria exposed to 60 *μ*M of Ag^+^ ions. In addition, we found that the responses of bacteria to Ag^+^ ions were heterogeneous. We quantified the oscillations of cell-length with power spectral density, which appeared different from that of bacteria growing in the absence of Ag^+^ ions. Furthermore, a model similar to the predator-prey argument was developed to understand the observed oscillations of cell-length upon exposure to Ag^+^ ions. This model not only successfully produced the oscillations but also explained the observed heterogeneity in the bacterial responses to Ag^+^ ions.

## Introduction

Due to the rising of antibiotic resistance of bacteria^1–4^, alternatives of commonly prescribed antibiotics have been gaining broad interest and attention in the past few years. Noble metals and their derivatives have been promising candidates, while silver ions (Ag^+^) and silver nanoparticles (AgNPs) are among the most well studied^5–30^. It has been extensively reported that Ag^+^ ions and AgNPs can effectively suppress the growth of, and kill, bacteria^5–7,13–15,17,19,22,24,26,28–30^, which might open new avenues to fighting against drug resistance of harmful microbes.

Although much effort has been made to investigate the antibiotic activity of Ag^+^ ions and AgNPs, the temporal resolution of these studies is lacking, as pointed out by Durán *et al*.^29^. Few studies exist for how Ag^+^ ions and AgNPs suppress the growth of, and kill, bacteria in real-time. In addition, whether bacteria are capable of developing resistance against Ag remains controversial^6^. While some claimed that it is difficult for bacteria to develop resistance against Ag due to Ag’s multi-modal antimicrobial activities^28,31^, other studies suggested the opposite^32–34^. For example, Graves *et al*. showed that *Escherichia coli* (*E. coli*) bacteria evolve rapidly and develop resistance against AgNPs^32–34^.

In an effort towards understanding Ag’s antimicrobial activity and mechanism, we previously examined in depth the growth of bacteria in the presence of Ag^+^ and AgNPs and found that the growth rate of the bacteria remained unaffected but the lag time of the bacterial growth was extended^6^. This observation led to a phenomenological model consisting of a “suppressed” state of bacteria caused by Ag^+^ ions and AgNPs. In this “suppressed” state, the bacteria struggle and defend themselves from Ag-induced damages and stresses^6^. In this model, a fraction of the bacteria are able to fight through the damages and stresses caused by Ag^+^ ions and AgNPs, going back to an “active” state and growing normally^6^. In contrast, another fraction of the bacteria are killed, entering a “dead” state^6^. Although this phenomenological model explains the experimental observations very well^6^, such battles between the bacteria and Ag-induced damages and stresses have not been directly visualized in real-time.

Here we report our study on the morphological dynamics of live bacteria when subjected to Ag^+^ ions using time-lapse microscopy. We observed that a large fraction of bacteria exposed to Ag^+^ ions exhibited oscillations in their cell-lengths (i.e., elongating and shrinking back and forth). In addition to the oscillatory bacteria, we found that the responses of bacteria to Ag^+^ ions are heterogeneous: some bacteria shrink their lengths, while others grow and divide after struggling through the stresses. We also quantified the oscillations of the cell-length with power spectral density, which is different from that of bacteria growing in the absence of Ag^+^ ions. Furthermore, we developed a model based on the predator-prey model for qualitatively understanding the observed oscillation of cell-length upon exposure to Ag^+^ ions. This model not only successfully produced the cell oscillation but also explained the observed heterogeneity in the bacterial responses to Ag^+^ ions.

## Results

### Oscillation of cell-length and heterogeneous response of bacteria upon exposure to Ag^+^ ions

**Figure 1:**
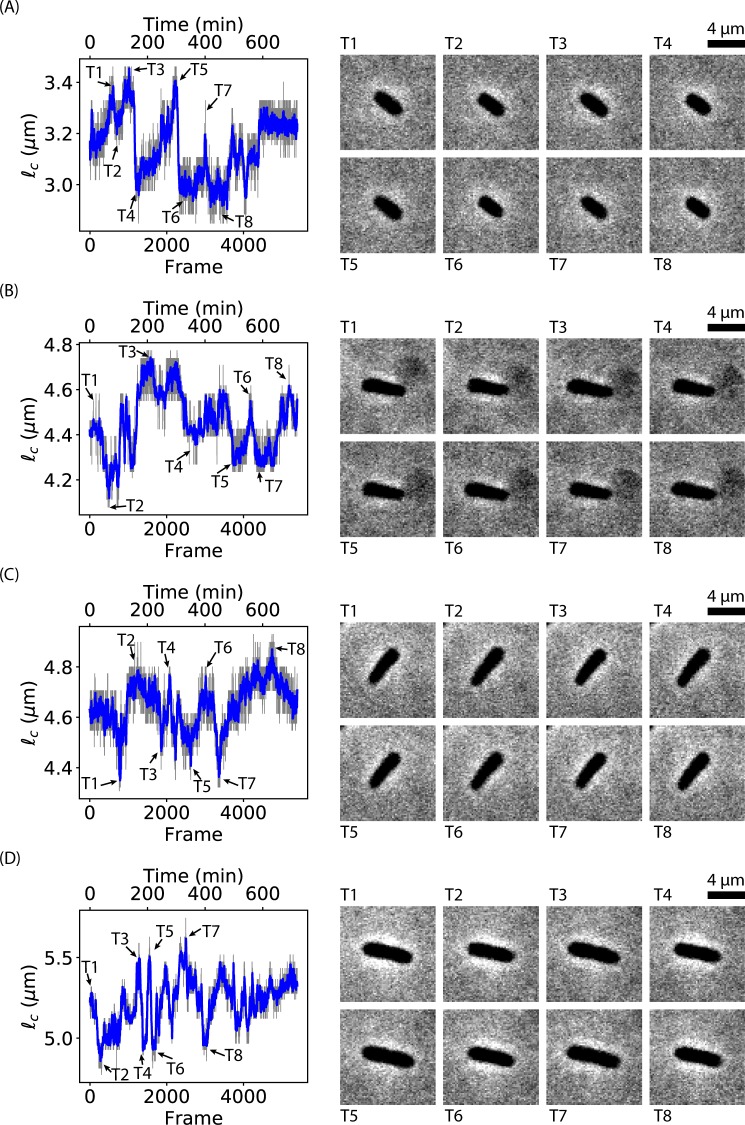
Oscillation in cell-length. (**A–D**) Cell-length as a function of frame number (time) from four examples of oscillatory bacteria in the presence of Ag^+^ ions. Representative peaks and valleys in the fluctuations were labeled by arrows and numbered as T1, T2, …, and T8. Images of the bacteria in the frames {*T*_*i*_} are shown in the right panels, extracted from (**A**) SI Movie M4, (**B**) SI Movie M5, (**C**) SI Movie M6, and (**D**) SI Movie M7.

Although it was reported previously that bacteria shrank their lengths when subjected to Ag^+^ ions^35^, temporal resolution was lacking in the previous studies^29^. We exploited time-lapse imaging to examine the dynamics of bacterial morphology after treating the bacteria with Ag^+^ ions (SI Movies M1 and M2). Most interestingly, we found that a large fraction of bacteria (24 out of 31 bacteria in SI Movie M1, or ~68% on average from replicated experiments) showed oscillations in their cell-length as a function of time. In other words, the lengths of bacteria increased and decreased back and forth during 12 hours in the presence of Ag^+^ ions. In contrast, a negative control showed that the bacteria grew and divided normally in the absence of Ag^+^ ions (SI Movie M3). To see the oscillations of cell-length in the presence of Ag^+^ ions more clearly, four individual oscillating bacteria were cropped out (SI Movies M4–M7) and their lengths as functions of time (or frame number) were quantified using ImageJ, MicrobeJ and/or Oufti (Fig. 1A–D, respectively)^36–39^. The raw data from MicrobeJ and/or Oufti (gray curves in Fig. 1) for the cell-length have been smoothed by convolving with a Hanning window with a window size of 11 frames (blue curves in Fig. 1)^40,41^. For each bacterium, eight frames at peaks/valleys in the length-vs-time curves were shown (T1 to T8, Fig. 1), from which visual differences in the cell-length can be observed. We note that these oscillations lasted for at least 12 hours in our experiments (Fig. 1). The magnitude (i.e., maximum - minimum) of the oscillations ranged from 0.4 *μ*m to 1.5 *μ*m, with a mean of 0.75 ± 0.27 *μ*m (mean ± standard deviation, sample size = 20).

**Figure 2:**
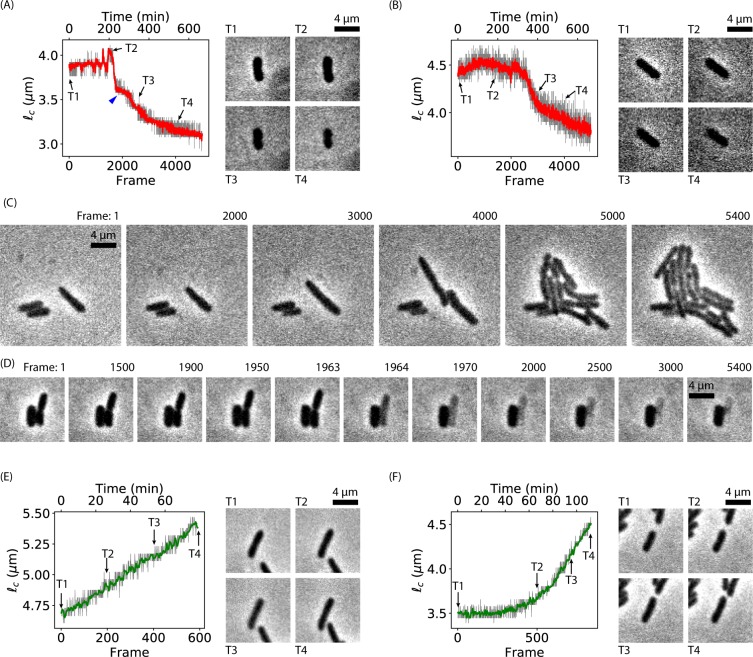
Heterogeneity of bacterial responses to Ag^+^ treatment. (**A,B**) Cell-length as a function of frame number (time) for two examples of shrinking bacteria in the presence of Ag^+^ ions. Representative frames were labeled by arrows and numbered as T1, T2, T3, and T4. Images of the bacteria in the frames {*T*_*i*_} are shown in the right panels, extracted from (**A**) SI Movie M8 and (**B**) SI Movie M9. (**C**) Frames of SI Movie M10 showing the growth of bacteria starting between Frame 2000 and 3000. (**D**) Frames of SI Movie M11 showing the burst of two cells happening between Frame 1950 and 2000. (**E,F**) Cell-length as a function of frame number (time) for two examples of growing bacteria in the absence of Ag^+^ ions (i.e., negative control). Representative frames were labeled by arrows and numbered as T1, T2, T3, and T4. Images of the bacteria in the frames {*T*_*i*_} are shown in the right panels, extracted from (**A**) SI Movie M13 and (**B**) SI Movie M14.

The observed oscillations of cell-length when subjecting bacteria to Ag^+^ ions are not artifacts due to the following reasons. First, the observations of oscillatory cell-length were reproducible. We repeated the experiments five times on different days and reproduced that bacteria treated with Ag^+^ ions exhibited oscillatory behavior. For example, SI Movie M2 was taken on a different day other than SI Movie M1. Second, the change in the cell size is unlike due to focusing/defocussing of the microscope, because an autofocus system was used during the time-lapse imaging of 12 hours. Third, we observed large heterogeneity in the bacterial responses to Ag^+^ ions (Fig. 2A–D). For example, apart from the oscillatory bacteria, some cells in the same field of view showed shrinkage (Fig. 2A,B, SI Movies M8 and M9). Among all the replicated experiments, ~31% of the bacteria eventually shrank. The shrinkage was previously reported although the dynamics was unknown^35^. Interestingly, ~55% of the shrinking bacteria showed step-wise behaviors, an example of which was highlighted by the blue arrow in Fig. 2A. In addition to the shrinking bacteria, ~7% of the bacteria grew and divided after oscillations (Fig. 2C, SI Movie M10), indicating that these bacteria have won the battle and overcome the antimicrobial effects posed by Ag^+^ ions. Furthermore, we observed that a small fraction (~2%) of the bacteria eventually exploded (Fig. 2D and SI Movie M11). The forth reason is that the oscillations of different bacteria were unsynchronized. An example is shown in SI Movie M12, where five bacteria were aligned well but the oscillations of these cells were independent to each other. Lastly, negative control experiments were performed with the same bacteria and microscope but in the absence of Ag^+^ ions (SI Movie M3), from which we observed monotonic growth of bacteria. For example, 100% of the initial ~120 bacteria in SI Movie M3 grew and divided normally. Most (~85%) of the quantified bacteria (sample size = 34) showed nearly linear increase in their cell-lengths (Fig. 2E, SI Movie M13, and SI Fig. S1A–E), although lagging was observed for a fraction of bacteria (~15%, Fig. 2F, SI Movie M14). The average of the normalized cell-length (${\ell }_{c}/{\ell }_{c0}$, where ${\ell }_{c0}$ is the cell-length of a bacterium in frame 0) for 16 bacteria with trajectories of at least 200 frames (including the lagging ones) was shown in SI Fig. S1F, clearly displaying the nearly linear growth of bacteria in the absence of Ag^+^ ions, consistent with results previously reported in the literature^42^. Therefore, all the evidence suggests that the observed oscillations of cell-length are actual.

We quantified the heterogeneity of the responses of bacteria to Ag^+^ ions by counting the number of bacteria in each category of responses. Out of the quantified 99 bacteria from replicated experiments, we observed that (1) 54% of the bacteria showed oscillations for 12 hours (i.e., no obvious growth or shrinkage at the end of the 12 hours), (2) 10% of the bacteria shrank after oscillations, (3) 21% of the bacteria shrank directly, (4) 7% of the bacteria grew and divided after oscillations or directly, (5) 2% of the bacteria exploded eventually, and (6) 6% of the bacteria stayed fairly constant. We speculate that the observed heterogeneity might result from (1) differences in the intrinsic sensitivity of the bacteria to Ag^+^ treatment, (2) variances in the local concentrations of Ag^+^ ions, and (3) differences in the rate of adaptation of bacteria to and/or resistance against Ag^+^ ions and Ag-induced damages and stresses. However, further studies are needed to determine the underlying mechanism of the observed heterogeneity.

**Figure 3:**
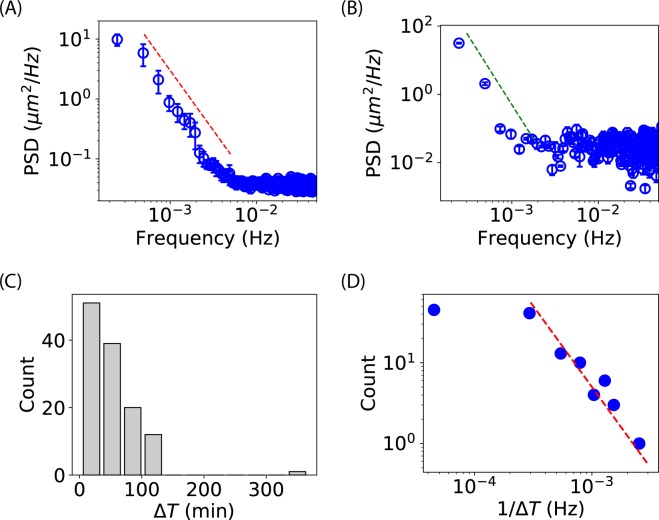
Quantification of cell-length curves. (**A**) Averaged PSD for the four unsmoothed oscillatory cell-length curves in Fig. 1. The red dashed line indicates a slope of −2 in the log-log scale. (**B**) Averaged PSD for the two unsmoothed growing cell-length curves in Fig. 2E,F. The green dashed line indicates a slope of −4 in the log-log scale. Error bars in (**A**,**B**) stand for standard error of the mean (SEM). (**C**) Histogram of the time interval Δ*T* between peaks (manually identified). (**D**) Histogram of the frequency 1/Δ*T* of peaks (manually identified). The red dashed line indicates a slope of −2 in the log-log scale.

We asked whether there exist any characteristic frequencies/periods in the oscillations of cell-length. The answer is negative when looking at the cell-length curves (Fig. 1). In addition, it appears that the oscillatory patterns of different bacteria are different (Fig. 1), suggesting the absence of characteristic oscillatory frequencies. Furthermore, we examined the power spectral density (PSD)^43^ of the cell-length curves using the Welch method (with a Hanning window and a length of segment of 512)^41,44^. The averaged PSD from the four unsmoothed oscillatory cell-length curves (Fig. 1) is shown in Fig. 3A, where the error bars stand for the standard error of the mean (SEM). We observed that the PSD did not display any characteristic peak. Instead, it was similar to the response of a low-pass filter. The slope of the power-law regime in the log-log scale is close to −2 (red dashed line in Fig. 3A). We also examined the PSD for the negative controls (i.e., the growing bacteria in the absence of Ag^+^ ions, Fig. 2E,F) and found that the power-law exponent is close to −4 (green dashed curve in Fig. 3B), different from the oscillatory cell-length curves. In addition, the power-law regime shifted to lower frequencies compared to the oscillatory cell-length curves. Lastly, to further confirm the absence of characteristic frequency/period in the oscillations of cell-length, we manually identified peaks in the cell-length curves (Fig. 1) and calculated the time intervals (Δ*T*) between peaks. The histogram of Δ*T* did not show obvious peaks (Fig. 3C), indicating the absence of characteristic periods once again. To compare the manual results with the PSD, we looked at the distribution of 1/Δ*T* (i.e., the “frequency”). Interestingly, a power-law with an exponent of −2 was observed around 10^−3^ Hz (Fig. 3D), consistent with power-law exponent from the PSD (Fig. 3A).

### Predator-prey-like model for understanding the oscillations of cell-length

**Figure 4:**
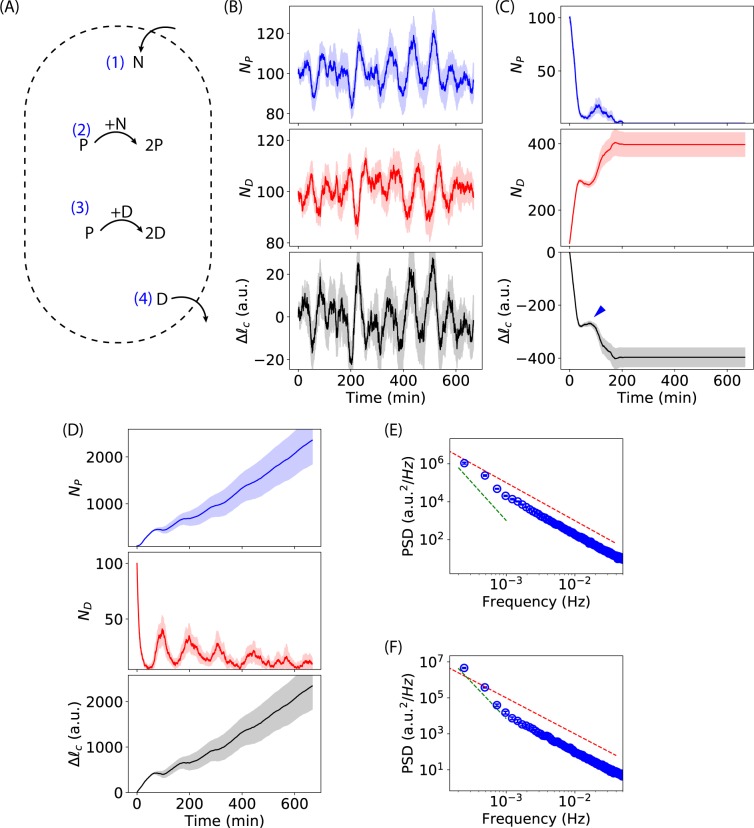
Predator-prey-like model. (**A**) Illustration of the components and dynamics of the system in our model. The “reactions” (1)–(4) in the system are described in the text. (**B**) Simulated oscillation of cell-length with simulation parameters (*r*_*i*_ = 0.1 s^−1^, *r*_*g*_ = 10^−5^ s^−1^, *r*_*d*_ = 10^−5^ s^−1^, and *r*_*o*_ = 10^−3^ s^−1^) and initial conditions (*N*_*N*0_ = 100, *N*_*P*0_ = 100, and *N*_*D*0_ = 100). (**C**) Simulated cell shrinkage with the same initial conditions and parameters as in (**B**) except *r*_*o*_ = 10^−4^ s^−1^. (**D**) Simulated cell growth with the same initial conditions and parameters as in (**B**) except *r*_*o*_ = 3 × 10^−3^ s^−1^. The dark lines in (**B–D**) are averages of 10 simulations, while the light areas stand for the standard errors of the means. (**E,F**) Power spectral densities of simulated cell-length curves with the same initial conditions and parameters as in (**B**,**D**). The red dashed line indicates a slope of −2, while the green dashed line indicates a slope of −4 in the log-log scale.

To understand the experimentally observed oscillations of cell-length after subjecting the bacteria to Ag^+^ ions, we developed a model based on the predator-prey argument^45,46^. Briefly, we consider a system composed of nutrients (*N*), positive growing components such as active proteins and other cellular products (*P*), and damaging components such as Ag^+^ ions, protein/DNA damages, and reactive oxygen species (*D*). The dynamics of this system is sketched in Fig. 4A, which shows the following four “reactions”.

1. The nutrients are taken into the bacteria from the growth medium with an intake rate of *r*_*i*_,
  $$\varnothing \mathop{\to }\limits^{{r}_{i}}N$$
2. The positive growing components are produced with a growth rate of *r*_*g*_ by consuming nutrients,
  $$P+N\mathop{\to }\limits^{{r}_{g}}2P$$
3. The positive growing components are damaged by the damaging components with a damaging rate of *r*_*d*_,
  $$P+D\mathop{\to }\limits^{{r}_{d}}2D$$
4. The damaging components are removed from the bacteria with an ousting rate of *r*_*o*_,

$$D\mathop{\to }\limits^{{r}_{o}}\varnothing $$

The first three “reactions” are straightforward, while the fourth one represents the battle of bacteria against Ag^+^ ions and Ag-induced damages and stresses. We note that removal of the damaging components are possibly due to multiple pathways. First, Ag^+^ ions could be pumped out of the bacteria by efflux pumps, which has been reported as a general mechanism of bacterial resistance against most toxic heavy metals^34,47,48^. A second pathway is degradation and repairing. Degradation of damaged proteins and repairing of damaged DNA are well-known means of quality control in bacteria^49,50^. Another possible way to remove the damaging components is that the bacteria might mutate and develop genotypic resistance against Ag^32^.

The corresponding differential equations for the number of the three species (*N*, *P*, and *D*) in the system are

1 $$\frac{d{N}_{N}}{dt}=+{r}_{i}-{r}_{g}{N}_{P}{N}_{N}$$

2 $$\frac{d{N}_{P}}{dt}=+{r}_{g}{N}_{P}{N}_{N}-{r}_{d}{N}_{P}{N}_{D}$$

3 $$\frac{d{N}_{D}}{dt}=+{r}_{d}{N}_{P}{N}_{D}-{r}_{o}{N}_{D}$$

The system was simulated using the Gillespie algorithm^51,52^. The simulations were run for a time duration of *T* = 4 × 10^4^ s, close to the duration of our experiments (12 hours, Fig. 1 and 2A–D). The initial conditions used for the simulations were *N*_*N*0_ = 100 (i.e., bacteria have a nutrient reservoir at the time of Ag^+^ treatment), *N*_*P*0_ = 100 (i.e., bacteria are not killed immediately by Ag^+^ ions and have remaining positive growing component), and *N*_*D*0_ = 100 (i.e., bacteria are partially damaged upon exposure to Ag^+^ ions). To compare the simulations with our measurements, we assumed that the cell length of bacteria is proportional to the difference between the positive growing components *P* and the damaging components *D*, i.e., ${\ell }_{c}\propto {N}_{P}-{N}_{D}$. Then the change in the cell length is indicated by ${\rm{\Delta }}{\ell }_{c}\propto {\rm{\Delta }}{N}_{P}-{\rm{\Delta }}{N}_{D}$. With *N*_*P*0_ = *N*_*D*0_ = 100, it is simplified to ${\rm{\Delta }}{\ell }_{c}\propto {N}_{P}-{N}_{D}$.

Oscillations of ${\rm{\Delta }}{\ell }_{c}$ (and ${\ell }_{c}$) were observed in simulations with appropriate parameters. The dynamics of *N*_*P*_, *N*_*D*_ and ${\rm{\Delta }}{\ell }_{c}$ from 10 simulations with *r*_*i*_ = 0.1 *s*^−1^, *r*_*g*_ = 10^−5^ *s*^−1^, *r*_*d*_ = 10^−5^ *s*^−1^, and *r*_*o*_ = 10^−3^ *s*^−1^ are shown in Fig. 4B, where the dark lines represent the averages of the repeated simulations and the lighter areas show the standard errors of the means (SEM). Oscillations of *N*_*P*_ and *N*_*D*_ were observed, similar to the populations of predators and preys in the predator-prey models^46^. Oscillatory fluctuations were also observed for the change in the cell-length, ${\rm{\Delta }}{\ell }_{c}$ (Fig. 4B). In addition, this predator-prey-like model also predicts shrinkage and growth of the bacteria, if different parameters are chosen. If the bacteria cannot remove Ag^+^ ions and Ag-induced damages quickly enough (i.e., lower ousting rate *r*_*o*_), the bacteria shrink their lengths. For example, when using *r*_*o*_ = 10^−4^ *s*^−1^ (but keeping the other parameters the same as those in Fig. 4B), bacterial shrinkage was observed (Fig. 4C). Interestingly, this model predicted that decreases in the cell-length might be step-wise (blue arrow for ${\rm{\Delta }}{\ell }_{c}$ in Fig. 4C), which was observed experimentally (blue arrow in Fig. 2A). In contrast, if the bacteria are capable of removing Ag^+^ ions and Ag-induced damages more quickly (i.e., higher ousting rate *r*_*o*_), cell growth was observed (Fig. 4D where *r*_*o*_ = 3 × 10^−3^ s^−1^).

Furthermore, we examined the power spectral density (PSD) of the simulated oscillatory cell-length curves. For this study, 10 simulations were ran for *T* = 4 × 10^5^ s with the parameters used in Fig. 4B. The averaged cell-length curve (represented by ${\ell }_{c}\propto {N}_{P}-{N}_{D}$) was computed for estimating the PSD by the Welch method (with a Hanning window and a length of segment of 512)^41,44^. The resultant PSD is shown in Fig. 4E, where the red and green dashed line indicate slopes of −2 and −4, respectively, in the log-log scale. We note that direct comparison for the magnitude of the PSD between the simulations and the experiments are not appropriate as the simulated cell-lengths were not actual lengths due to the unknown proportionality constant. Instead, we focused on comparing the slopes in the PSD data. We observed that the PSD of the simulated oscillating cell-length curves has a slope of −2, consistent with the experimental measurements (Fig. 3A). On the other hand, we found that a flat region at higher frequencies in the experimental result (Fig. 3A) was not present in the PSD of the simulated curves (Fig. 4E). A possible reason is that the flat region in the experimental measurements was due to experimental noises. In contrast, the PSD for simulated growing bacteria (Fig. 4F, with the same parameters as used in Fig. 4C but *T* = 4 × 10^5^ s) showed a kink around 10^−3^ Hz. The slope in the PSD for growing bacteria was −4 in the log-log scale at low frequencies (<10^−3^ Hz), consistent with the experimental result (Fig. 3B).

**Figure 5:**
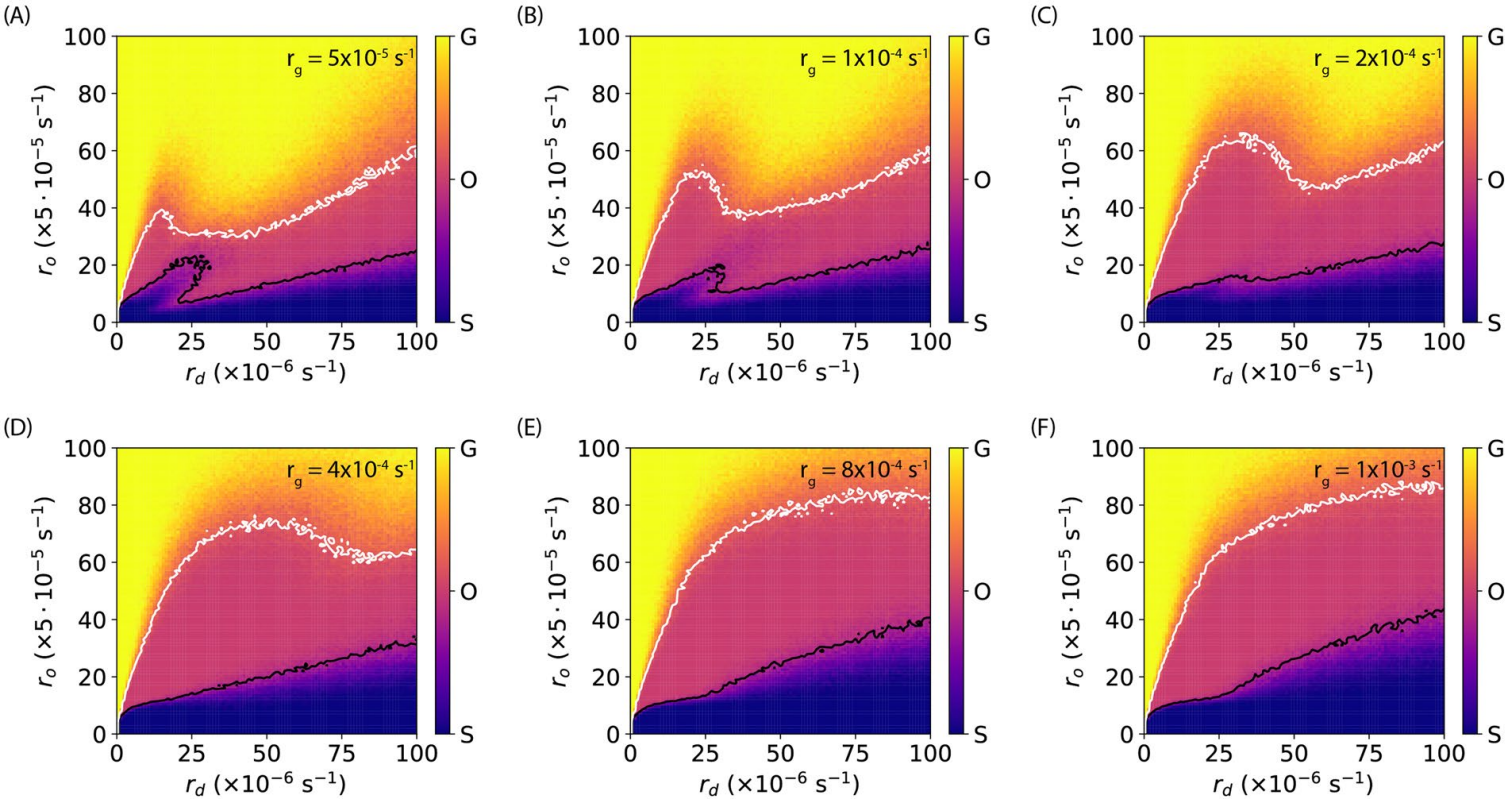
Phase diagrams (the ousting rate *r*_*o*_ *vs*. the damaging rate *r*_*d*_) of our model at different growth rates, (**A**) *r*_*g*_ = 5 × 10^−5^ s^−1^, (**B**) *r*_*g*_ = 1 × 10^−4^ s^−1^, (**C**) *r*_*g*_ = 2 × 10^−4^ s^−1^, (**D**) *r*_*g*_ = 4 × 10^−4^ s^−1^, (**E**) *r*_*g*_ = 8 × 10^−4^ s^−1^, and (**F**) *r*_*g*_ = 1 × 10^−3^ s^−1^. The white and black curves in (**A–F**) correspond to contour levels of 1.2 and 0.8, respectively. The legends on the color bars read as the following: G = growth, O = oscillation, and S = shrinkage.

Lastly, we examined the phase diagrams of our model by varying the three rates, *r*_*g*_ ∈ [0, 10^−4^] s^−1^, *r*_*d*_ ∈ [0, 10^−4^] s^−1^, and *r*_*o*_ ∈ [0, 5 × 10^−3^] s^−1^, while keeping the initial conditions and the intake rate constant (*N*_*N*0_ = *N*_*P*0_ = *N*_*D*0_ = 100 and *r*_*i*_ = 0.1 s^−1^). For each set of parameters (*r*_*g*_, *r*_*d*_, *r*_*o*_), 100 simulations were run for *T* = 4 × 10^4^ s. For each simulation, the type of the bacterial response was determined by comparing the difference between the end-point values of *N*_*P*_ and *N*_*D*_ with a threshold *N*_*th*_: growth if (*N*_*P*_−*N*_*D*_) > + *N*_*th*_, oscillation if −*N*_*th*_ ≤ (*N*_*P*_−*N*_*D*_) ≤ +*N*_*th*_, and shrinkage if (*N*_*P*_−*N*_*D*_) < −*N*_*th*_. Then the probability of the three types of responses (*p*_*g*_, *p*_*o*_, *p*_*s*_) were estimated from the 100 simulations. To report different types of responses in the same phase diagrams, we computed the weighted averages, $\bar{p}=G\times {p}_{g}+O\times {p}_{o}+S\times {p}_{s}$, where *G* = 2, *O* = 1, and *S* = 0. If the weighted average is close to *O* = 1, the most probable response would be oscillation. The phase diagrams (*r*_*o*_ *vs. r*_*d*_) of our model at different *r*_*g*_ values with *N*_*th*_ = 150 are shown in Fig. 5, where the white and black lines represent the contour levels of 1.2 and 0.8 (close to *O* = 1), respectively. As seen from the phase diagrams (Fig. 5), at low ousting rates (*r*_*o*_), it is most likely that bacteria would shrink for most damaging rates (*r*_*d*_). With a given damaging rate (*r*_*d*_), the most probable response of the bacteria changes from shrinkage to oscillation and then to growth, as the ousting rate *r*_*o*_ increases. This result is expected because a higher ousting rate corresponds to higher resistance of bacteria against Ag. It is noted that, although the phase diagrams were qualitatively similar, quantitative differences were observed for different growth rates *r*_*g*_ (Fig. 5), suggesting that *r*_*g*_ also plays a role in the bacterial response in our model. We also note that the phase diagrams with different thresholds gave similar results as shown in SI Fig. S2 where *N*_*th*_ = 100 or *N*_*th*_ = 200.

## Discussions

In this study, we used time-lapse microscopy to examine the morphological dynamics of live *E. coli* bacteria when subjected to Ag^+^ ions at 60 *μ*M. We observed that approximately 68% of the bacteria exposed to Ag^+^ ions elongated and shrank back and forth, clearly displaying oscillatory behaviors. In addition, we found that the bacterial responses to Ag^+^ ions are heterogeneous: in addition to the oscillating ones, ~21% of the bacteria simply shrank their lengths, while ~7% of them won the battle against Ag^+^ ions and eventually grew and reproduced. We quantified the oscillations of the cell-length by examining the power spectral density (PSD), which showed a power of −2. In contrast, the PSD of growing cells in the absence of Ag^+^ ions displayed a power of −4. Furthermore, a predator-prey-like model was developed to qualitatively understand the observed oscillation of the cell-length upon exposure to Ag^+^ ions. This model not only produced the cell oscillations but also explained the observed heterogeneity in the bacterial responses to Ag^+^ ions. The PSDs predicted by our model were partially consistent with the experimental results. Lastly, we examined the phase diagrams of our model.

The current study provides an experimental explanation for our previous observations that the lag time of the bacterial growth was significantly elongated in the presence of Ag^+^ ions at moderate concentrations^6^. The oscillations of the cell-length indicate that the bacteria fight against the stresses and/or other negative effects caused by Ag^+^ ions, and that bacteria attempt to adapt to the Ag^+^ environment during the elongated lag time. In addition, the current results suggest that the oscillatory stage of the bacteria corresponds to the “suppressed” state of bacteria in our previous phenomenological “SAD” model^6^, or the active but nonculturable (ABNC) state observed by Jung *et al*.^53^. In addition, our data showed that a fraction of the bacteria are able to grow again after several cycles of oscillations, supporting the assumed transition from the “suppressed” state to the “active” state in our previous “SAD” model^6^.

Oscillations play important roles in many dynamic cellular processes in both eukaryotes and prokaryotes^54^. For example, Min proteins oscillate in bacteria from one pole to the other, helping the bacteria to divide at appropriate time and place^54–56^. In addition, Tanouchi *et al*. observed that a subpopulation of bacteria might exhibit transient oscillations in cell size in long time scales with periods of several generations^57^. However, to our knowledge, oscillations of the cell-length of individual bacteria in short time scales were observed for the first time. An interesting question is whether and how these oscillations share common features and/or general underlying principles.

The assumption that Ag^+^ ions and Ag-induced damages are removed from the bacteria is essential in our model (Fig. 4A). This assumption implies that the bacteria possess certain resistance against Ag. However, it is worthwhile to emphasize that this assumption has no implication on whether the bacteria mutate and develop Ag-resistances. It is well known that endogenous resistance of bacteria to most toxic heavy metal is based on energy-dependent efflux of ions by membrane proteins that function as ATPases or chemiosmotic antiporters^47,48^. For example, Franke *et al*. reported that the *cus* determinant (*cusS* and *cusCFBA*) on the chromosome of *E. coli* (previously known as the ybcZ-ylcA ylcBCD-ybdE region) is responsible for the bacterial resistance to Cu and Ag, because deletion of these genes resulted in sensitive mutants than the wild type^58^. A later systematic study by Ivask *et al*. suggested that the several genes in the *cus* determinant were among the 35 common *E. coli* mutants that were sensitive to Ag^+^ ions and AgNPs^30^. In addition, outer membrane proteins (e.g., *ompR*, *ompC*, *ompF*, and *ompA*) have also been shown to contribute to the bacterial resistance against Ag; these proteins are essential for the formation of pores to allow passive diffusion of small molecules across the outer membrane^59,60^. Due to their importance in removing Ag^+^ ions, these efflux pumps could be promising candidates accounting for the molecular mechanism of the observed oscillations of cell-length. On the other hand, our model does not exclude the possibility of development of further resistance of bacteria against Ag^+^ ions, which have been observed in previous studies. For example, mutations in *ompR* and *cusS* resulted in more Ag-resistant *E. coli* strains^32,60^. Mutations in other genes might also lead to greater fitness than the wild type in Ag-containing environments^32^. It is noted that previous studies showed that mutations associated with Ag-resistance accumulated before 100 generations after Ag-treatment (>15 days)^32^; however, it is unclear whether mutations were present in our experiments, which lasted for only 12 hours. Future experiments with morbidostats^61,62^ would help to address these questions.

The phase diagrams of our model suggested that the response of bacteria to Ag^+^ ions relies on the relation between the ousting rate *r*_*o*_ and the damaging rate *r*_*d*_. Our model suggests that, at a given concentration of Ag^+^ ions (corresponding to a given damaging rate *r*_*d*_), the ability of the bacteria to remove Ag^+^ ions and Ag-induced damages determines the fate of the bacteria. Therefore, our model predicts that deletions of the efflux pumps and pores (i.e., *cusCFBA* and *ompCF*) would result in lower ousting rates *r*_*o*_ and lead to more sensitive and vulnerable strains compared to the wild type. This prediction is consistent with experimental results previously reported in the literature^30,58^. In addition, our model predicts that mutations in the *cus* and *omp* systems that result in a higher ousting rate of Ag^+^ would provide the bacteria stronger resistance, which could be experimentally tested using mutants in future studies.

Lastly, the observed battles between bacteria and Ag^+^ ions and Ag-induced damages/stresses suggest that care must be taken with regards to using Ag as antimicrobial agents, and unintentional exposure of microbes to Ag should be minimized.

## Materials and Methods

### Bacterial strain and growth

The *E. coli* strain used in this study is JW1225 of the Keio collection^59^ (purchased from the Yale *E. coli* Genetic Stock Center) transformed with a plasmid encoding *hns-meos* fusion gene^63^. The resultant strain (named K12 Δhns/pHNS-mEos3.2) expresses H-NS proteins fused to mEos3.2 photo-switchable fluorescent proteins^63,64^ and has kanamycin and chloramphenicol resistance^63,65^. This strain has been used in our other studies^65^, including several studies on the antibiotic activities of Ag^+^ ions and AgNPs^66^.

The bacteria were grown at 37 °C overnight in Luria broth (LB) medium supplemented with kanamycin and chloramphenicol. On the second day, the overnight culture was diluted by 50 to 100 times into fresh medium so that the OD 600 was 0.05. The fresh cultures were again grown at 37 °C until the OD 600 reached ~0.3, followed by adding Ag^+^ ions into the culture at a final concentration of 60 *μ*M. Then 10 *μ*L of the bacteria were transferred to a 5 mm × 5 mm agarose pad (1% in the growth medium supplemented with 60 *μ*M Ag^+^ ions in the form of AgNO_3_ solutions). For negative controls, Ag^+^ were omitted in both the liquid medium and the agarose pad. The agarose pad with bacteria was flipped and attached to a clean coverslip (cleaned with sonication in detergent, 1 M NaOH, 100% ethanol, and ultra-pure water sequentially). A chamber was then constructed by sandwiching a rubber o-ring between the coverslip and a microscope slide. The chamber was sealed using epoxy glue and then mounted for time-lapse imaging.

### Time-lapse microscopy

Time-lapse imaging experiments were carried out at room temperature on an Olympus IX-73 inverted microscope in the phase contrast mode equipped with an Olympus 100X N.A. = 1.25 objective and an EMCCD (Andor Technology). The effective pixel size of acquired images was 160 nm. The microscope and data acquisition were controlled by Micro-Manager^67^. A CRISP autofocus system (Applied Scientific Instrumentation, USA) was used to keep the sample focused during the time-lapse imaging. For the acquisition of the time-lapse movies, the exposure time for each frame was 30 ms, and the time interval between frames was 8 s.

### Quantification of cell-length from time-lapse movies

The acquired time-lapse movies were corrected for x/y drift by cross-correlation using the scikit-image python module^68,69^ and analyzed in ImageJ^36,37^. First, the movies were inverted, followed by removing background using the rolling ball method (with a radius of 10 pixels)^70^. After smoothing, the images were inverted back, followed by cropping out movies for individual cells with ImageJ. For the movies of individual cells, MicrobeJ (an ImageJ plugin) and/or Oufti were applied to obtain the cell-length as a function of frame number^38,39^. The default algorithm (i.e., modified IsoData) was used for thresholding in MicrobeJ, while the smoothed option was turned on for detecting the bacteria edges^38^. The cell-lengths were outputted after running MicrobeJ/Oufti on the movies of individual cells.

## Supplementary information

Supplementary Information

SI Movie M1

SI Movie M2

SI Movie M3

SI Movie M4

SI Movie M5

SI Movie M6

SI Movie M7

SI Movie M8

SI Movie M9

SI Movie M10

SI Movie M11

SI Movie M12

SI Movie M13

SI Movie M14
