# Supplementary material for: Silver ions cause oscillation of bacterial length of Escherichia coli
Source: Sci Rep. 2019 Aug 13;9:11745. doi: 10.1038/s41598-019-48113-4 (PMC6692348; doi:10.1038/s41598-019-48113-4)
Supplement: Supplementary file 1 — Supplementary Information [file 41598_2019_48113_MOESM1_ESM.pdf]

Silver ions cause oscillation of bacterial length of *Escherichia coli*

Venkata Rao Krishnamurthi[1], **Jingyi Chen**[2,3], **and Yong Wang**[1,3,4,*]

[1]Department of Physics, University of Arkansas, Fayetteville, AR 72701

[2]Department of Chemistry and Biochemistry, University of Arkansas, Fayetteville, AR 72701 [3]Microelectronics-Photonics Graduate Program, University of Arkansas, Fayetteville, AR 72701

[4]Cell and Molecular Biology Program, University of Arkansas, Fayetteville, AR 72701

[*]To whom correspondence should be addressed: yongwang@uark.edu (Y.W.).

Supplementary Information

2 figures and 14 movies.

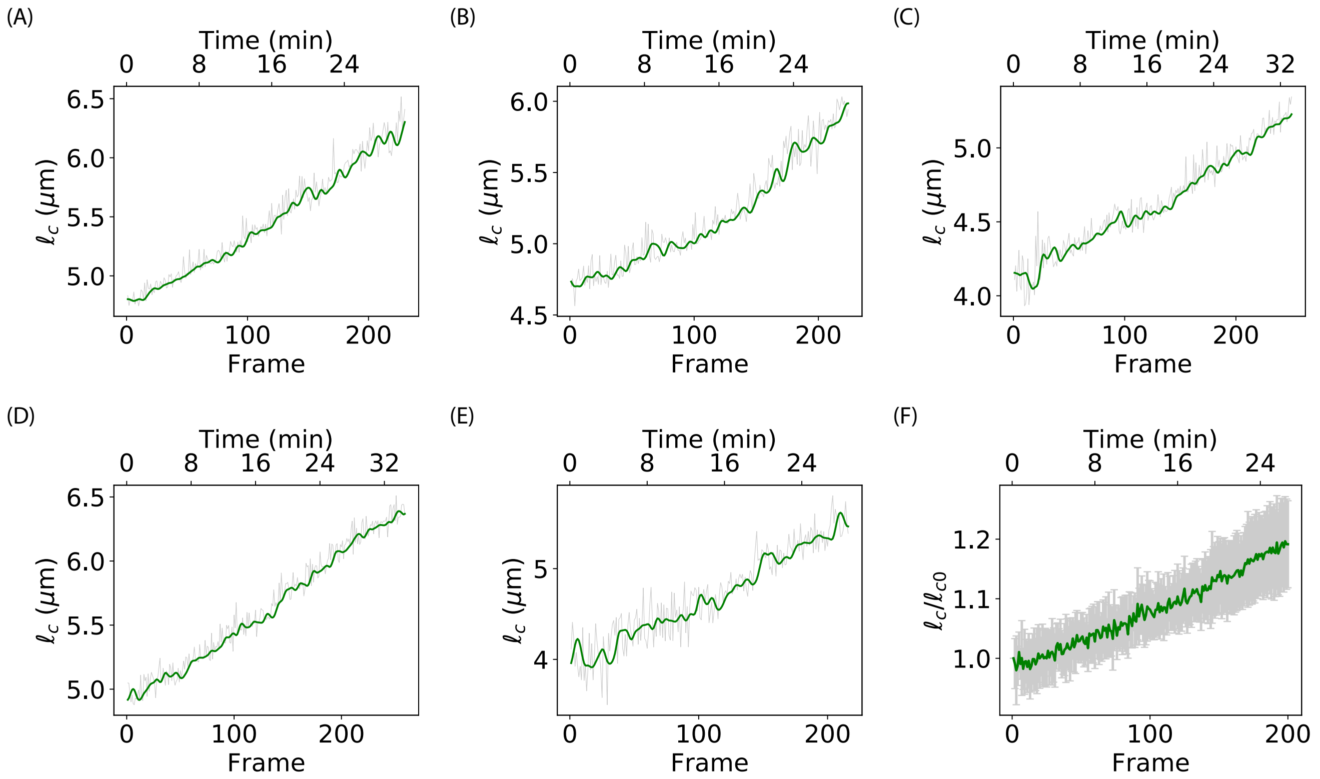

Figure S1. Cell-length as a function of frame number (time) for more examples of growing bacteria in the absence of Ag^+ ions (i.e., negative control). (A-E) Cell-length (ℓ_c) of individual bacteria. The gray curves are raw data, while the green curves are smoothed ones. (F) Average of normalized cell-length, ℓ_c/ℓ_{c0}, from 16 bacteria, where ℓ_{c0} is the cell-length at frame 0. Error bars represent the standard deviation.

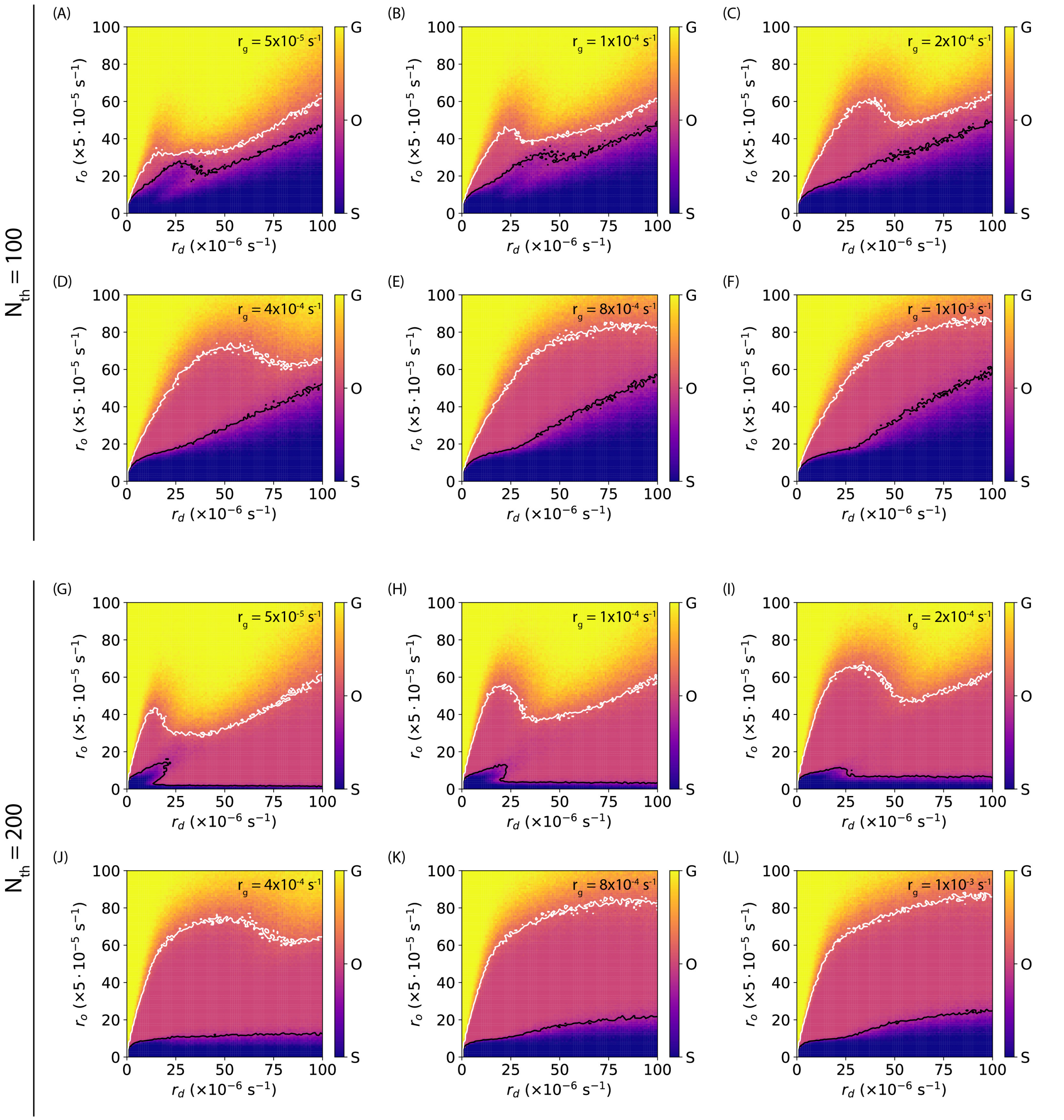

Figure S2. Phase diagrams (the ousting rate r_o *vs.* the damaging rate r_d) of our model at different growth rates, (A,G) $r_g = 5 \times 10^{-5}$ s^{-1}, (B,H) $r_g = 1 \times 10^{-4}$ s^{-1}, (C,I) $r_g = 2 \times 10^{-4}$ s^{-1}, (D,J) $r_g = 4 \times 10^{-4}$ s^{-1}, (E,K) $r_g = 8 \times 10^{-4}$ s^{-1}, and (F,L) $r_g = 1 \times 10^{-3}$ s^{-1}. The white and black curves in (A–L) correspond to contour levels of 1.2 and 0.8, respectively. The legends on the color bars read as the following: G = growth, O = oscillation, and S = shrinkage. The threshold N_{th} is 100 for (A–F), and 200 for (G–L).

SI Movie M1. Time-lapse movie of bacteria in the presence of 60 μM Ag^+ ions. Speed up: 10,000x. Total acquisition time: 12 hours.

SI Movie M2. Time-lapse movie of bacteria in the presence of 60 μM Ag^+ ions, measured on a different day. Speed up: 10,000x. Total acquisition time: 12 hours.

SI Movie M3. Time-lapse movie of bacteria in the absence of Ag^+ ions (i.e., negative control). Speed up: 10,000x. Total acquisition time: 6 hours.

SI Movie M4. Time-lapse movie of a single bacterium showing oscillatory cell-length in the presence of 60 μM Ag^+ ions (Fig. 1A, cropped from SI Movie M1). Speed up: 10,000x. Scale bar: 4 μm.

SI Movie M5. Time-lapse movie of a single bacterium showing oscillatory cell-length in the presence of 60 μM Ag^+ ions (Fig. 1B, cropped from SI Movie M1). Speed up: 10,000x. Scale bar: 4 μm.

SI Movie M6. Time-lapse movie of a single bacterium showing oscillatory cell-length in the presence of 60 μM Ag^+ ions (Fig. 1C, cropped from SI Movie M1). Speed up: 10,000x. Scale bar: 4 μm.

SI Movie M7. Time-lapse movie of a single bacterium showing oscillatory cell-length in the presence of 60 μM Ag^+ ions (Fig. 1D, cropped from SI Movie M1). Speed up: 10,000x. Scale bar: 4 μm.

SI Movie M8. Time-lapse movie of a single bacterium showing shrinking cell-length in the presence of 60 μM Ag^+ ions (Fig. 2A, cropped from SI Movie M1). Speed up: 10,000x. Scale bar: 4 μm.

SI Movie M9. Time-lapse movie of a single bacterium showing shrinking cell-length in the presence of 60 μM Ag^+ ions (Fig. 2B, cropped from SI Movie M2). Speed up: 10,000x. Scale bar: 4 μm.

SI Movie M10. Time-lapse movie of bacteria showing cell-growth and division in the presence of 60 μM Ag^+ ions (Fig. 2C, cropped from SI Movie M2). Speed up: 10,000x. Scale bar: 8 μm.

SI Movie M11. Time-lapse movie of a single bacterium showing cell shrinkage and explosion in the presence of 60 μM Ag^+ ions (Fig. 2D, cropped from SI Movie M1). Speed up: 10,000x. Scale bar: 4 μm.

SI Movie M12. Time-lapse movie of several bacteria showing asynchronized oscillation in the presence of 60 μM Ag^+ ions (cropped from SI Movie M2). Speed up: 10,000x. Scale bar: 8 μm.

SI Movie M13. Time-lapse movie of bacteria in the absence of Ag^+ ions (Fig. 2E, cropped from SI Movie M3). Movie length: 80 min. Speed up: 10,000x. Scale bar: 4 μm.

SI Movie M14. Time-lapse movie of bacteria in the absence of Ag^+ ions (Fig. 2F, cropped from a repeated experiment of SI Movie M3). Movie length: 112 min. Speed up: 10,000x. Scale bar: 4 μm.
